# Career Guidance and Counseling for University Students in China

**DOI:** 10.1007/s10447-012-9151-y

**Published:** 2012-04-01

**Authors:** Vincy Jing Sun, Mantak Yuen

**Affiliations:** The University of Hong Kong, Pokfulam Road, Hong Kong, China

**Keywords:** Career guidance, Career counseling, University students, China

## Abstract

In recent years, various forms of career guidance and career counseling have become more prominent and better serviced in most universities throughout the world. Such services are obviously to the benefit of the students themselves and for society. After an initially slow start, researchers and practitioners in China have now begun to focus on the localization of guidance and counselling theory and strategies in order to match more exactly actual employment situations in different regions of the country. This should result in a service that meets students' needs more effectively. Using mainly core literature examining the context of career guidance and counseling in China from 2001 to the present, this paper elaborates on the current situation and summarizes the progress that has been made. The authors detail the content, implementation, problems that exist, and ways of improving projects of this kind in Chinese universities. Conclusions and suggestions for further research on career guidance and counseling are provided.

## Introduction

Theories of career guidance and counseling that exist in China were mainly adopted from Europe and the United States, particularly in the period since 1990. However, one institution in China, Tsinghua University, actually started work in this field as early as 1916, and established a vocational guidance committee in 1923. This initiative can probably be regarded as the beginning of career guidance in China (Liu 2006). More recently, the country has been developing rapidly and there has been a significant increase in the need for career guidance. At a national level, new policies have been issued and implemented, and there has been expansion in the provision of training courses on careers matters for teachers. An increase in the publishing of teaching materials for career guidance curricula is also evident (Ma 2009).

Over the years, many career guidance centers have been set up in universities, accumulating some useful experiences in implementation of career guidance and counseling (Li 2002; Ma 2009). The following sections summarise some of the significant achievements and the state of current research on career guidance in China.

In reviewing the Chinese literature, the terms *zhiye guihua* (career planning) or *jiuye zhidao* (employment guidance) are used when referring to career guidance, career education and vocational guidance. Wang (2008) defined the difference between these two terms in the Chinese educational context, with *jiuye zhidao* (employment guidance) being concerned with issues related to finding jobs for prospective graduates. Issues addressed in this domain typically include: familiarization with employment policy, providing employment information, coaching on resumé preparation, and developing interview skills. *Zhiye guihua* (career planning), on the other hand, means guidance given to students according to their personal circumstances to assist them in planning for lifelong career development. Here, typically, students are made aware of the options available to them in the world of work, and they are led to realize the many important decisions that have to be made. Relevant topics are presented through the career guidance curriculum and via career counseling. Career assessment is also included.

In the situation existing at present in most Chinese universities, employment guidance still dominates over career planning (Wu 2008). Effective career planning is implemented in only a few universities, but currently other colleges and universities are beginning to make significant efforts to extend their employment guidance and counseling more in the direction of career planning (Bian 2008). In the complex and evolving situation existing in China, it is insufficient to address all aspects of career development by using only employment guidance strategies or by dealing only with career planning - both elements are required. For convenience, in this paper the term ‘career guidance’ will be used to embrace both ‘job-hunting skills’ and longer-term career planning.

## Recent and Current Employment Situation

As a result of national policy (State Council of China 1999), Chinese universities began to expand their enrolments in 1999, and this has ultimately created many problems in the current job market. The average rate of increase of new graduates in every year has been 30% since 2002, so graduating students have faced more and more severe employment difficulties (Geng 2007). In 2008, the total number of national university graduates reached 5.59 million; but according to official statistics the initial employment rate of graduates is only 70%, so there would be at least 1.7 million university graduates faced with a problem in gaining employment (Gao 2009). Indeed, difficulty getting a job has emerged as an ongoing focus of personal concern and anxiety, leading to an increased need for career counseling services (Wu 2008).

According to Gao (2009) and Wu (2008), the problem of employment for graduates is exacerbated by many factors, an important one of which is that they have limited long-term career planning and personal goal-setting strategies. They also lack awareness of the knowledge and competencies required in specific jobs. When faced later with employment demands, they generally exhibit such psychological problems as confusion, anxiety and panic.

## Significance of Career Guidance

Wen (2009) interpreted the significance of career guidance in university contexts from two perspectives, (i) the needs of students, and (ii) development of the university. For students, career planning can help them set personal goals and decide upon current and future directions. Such career guidance is helpful to them in overcoming any misunderstandings in choosing careers, in selecting their study paths, and in identifying their potential strengths to enhance their competitiveness for positions. Effective career guidance provides guidelines with a long-term vision for career planning, from which students can benefit by realizing their true potential in life (Guo 2009).

For universities, career guidance can help promote necessary reforms in teaching and can improve employment rates for their graduates, thus enhancing the reputation of a university. Solving the problem of employment is not only related to university students and their families, but also to the universities’ reputations, and even to the country's politics, construction of the economy, and maintaining a harmonious society (Li and Ye 2001).

## Current Situation and Results of Research

Despite its very early start in Tsinghua University, career guidance and counseling within most universities in China is still at a fairly elementary stage. This is reflected in the fact that Chinese papers and articles on career guidance have been mostly written after 2004. Key words searched in the Chinese journal data-base in relation to this study were ‘career guidance,’ ‘employment guidance’ and ‘career planning.’ It was found that there were few journals using ‘career guidance’ in titles or descriptors between 1979 and 2001. Within the decade from 2001 to 2011, approximately 250 papers about career guidance for university students’ were published in Chinese core journals. Of these, nearly 200 were written after 2005. It is almost impossible to find any paper on this issue in China written before 2001. This suggests that research on career guidance in China started late, but then developed very rapidly. In the material that does exist, the content is almost entirely about ‘employment guidance’ in universities, with just a few writers addressing career guidance in high schools (e.g., Liu and Tian 2008).

Fang and Tan (2010) claimed that papers on career counseling for university students in China are even fewer in number, and generally lack practical strategies or data from empirical studies. Articles tend to be confined to investigations of students' demand for career counseling (e.g., Zhao and Shen 2008), analysis of existing conditions in career guidance (e.g., Yu 2002), and describing the duties of career counselors (e.g., Fang and Tan 2010).

In the areas of both guidance and counseling, the content of many published papers mainly describes possible provisions, often based on models from other countries (e.g., Li 2002), identifies potential problems (e.g., Lan and Wang 2010), and suggests possible solutions (e.g., Yu 2008). There is very little reported research about the combination of career planning and selecting appropriate courses, or examples of empirical research on career guidance or counseling strategies. This situation suggests that the theoretical basis for career guidance in China needs to be strengthened (Liu and Li 2007). On the other hand, there is evidence in some reports that career guidance and counseling endeavours in some Chinese universities appear to have achieved very good results (e.g., Yu 2002), and these examples could provide a reference model for other universities in China to emulate (Wan and Wang 2006).

In addition to presenting foreign career theories, a number of papers focus on the current situation regarding career guidance in Chinese universities in different regions of China (e.g., Fang 2007; Zhou 2008). These studies tend to highlight a need to develop an *indigenous* career theory (or theories) to suit the actual situations existing in those regions.

## Implementation of Career Guidance

In terms of the methods of career guidance currently in operation, over 90% of 16 universities investigated in Beijing have open ‘employment guidance courses’ for introducing job-hunting skills and employment policy (Liu 2006). Other methods of delivering career guidance content to students include *ad hoc* lectures, campus recruitment fairs, and individual counseling as required. Only 25% of universities in Beijing provided career assessment services, and in Zhejiang Province, only three of the surveyed 21 universities provided such assessment (Hong 2007). At the present time, career guidance in universities is mostly delivered via ‘career centres,’ and through departmental career tutors (Long and Song 2007). However, many practitioners consider this traditional model usually fails to satisfy the many needs that students have for career guidance and career counseling.

To change the traditional mode of vocational guidance, some writers (e.g., Long and Song 2007; Sun 2009) suggest developing more comprehensive career guidance programmes. At present, a comprehensive approach to employment guidance has begun mainly in Beijing Normal University, Shanghai Jiaotong University, Renmin University of China, Fudan University and Guangxi University (Yu 2002). For instance, the model of comprehensive career planning in Beijing Normal University conducts preliminary guidance about "How to be successful" for freshman, all-round student development education for sophomores, guidance on vocational selection in junior years, with guidance for employment policies and techniques for application in the senior year. In Anhui Industry University, a ‘three-direction education model’ comprising (i) ‘education for direction of majors’ when students enter university, (ii) ‘education for situation awareness’ while at university itself, and, finally (iii) ‘education for choosing careers’ when students graduate, operates. So far, the effects of these new models appear to be positive (Yu 2008).

The approach to career guidance needs to be flexible and responsive to students’ needs and interests, and to local conditions. It is necessary to create a network of resources and tools that make career guidance more in-depth, relevant and accessible. One example of this is the *Occupational Information Network* in the United States (Peterson *et al*. 2001). Another example is an on-line course created in Fudan University in China. Students at Fudan can view 50 different video sessions as part of a career guidance course on-line by entering their individual login names and passwords. This on-line course is in four parts: career assessment, vocations introduction, curriculum study, and career counseling (Sun 2009). Tianjin Normal University provides a good example of using software applications for vocational assessment and planning. That establishment uses *Careersky* software to offer self-service for students in career education. The *Careersky* online career planning system is the most widely used and appears to be reasonably effective (Beisen Career Assessment 2007; Sun 2009).

Based on the problem of insufficient teaching personnel to service the area of career guidance, some universities have built independent learning channels for students, such as a website providing relevant information on career matters, making available appropriate print resources free of charge, and organizing relevant forums (Zhao and Shen 2008).

Yan (2008) reported that ‘experiential-style guidance’ is used in some universities, including simulated job interviews and the provision of firsthand vocational experiences. Internships and work fellowships in companies can also provide valuable opportunities for university students to observe at firsthand and practice the jobs in which they may be interested. Naturally, providing this form of off-campus experiential learning is not easy to arrange for large numbers of students. It also relies heavily on the willingness of local companies and worksites to cooperate and provide placements.

## Problems Existing in Career Guidance

At the moment there appears to be too little theory underpinning most of what universities and colleges attempt do in the way of career guidance. The institutions do not seem to establish career planning systems on any scientific or proactive basis, but instead respond to needs in an *ad hoc* manner, as and when they arise. The core duties of most career guidance centers are confined to providing information and processing employment procedures, such as registering employment whereabouts, guidance on signing contracts, and employment statistics. The service mostly emphasizes introduction to employment policy and analysis of the current employment situation. Professional career counseling is rarely provided automatically to all students; and even some counseling services that are offered are not individualized and focused enough to satisfy students' needs (Li 2009). There is a lack of well-structured and purposeful guidance to assist students’ career planning in a practical and personalized way.

Since there is still a scarcity of teaching materials and teachers’ texts designed specifically to match existing Chinese employment contexts, it is usual for universities to copy Western ideas. While some general principles may hold true in both cultures, Western policies and practices do not necessarily mesh with Chinese national conditions (Zhao and Shen 2008). Similarly, assessment tools relevant for career guidance in China have basically adopted three authoritative Western theories and measurement scales, namely, *Holland's Vocational Interest Theory* (Holland 1959), *Myers-Briggs Type Indicator* (Myers 1962), and *Edgar Schein’s Career Anchor* (Schein 1978). Due to the cultural differences between the West and China such assessment tools often lack local relevance for Chinese realities; and the data they produce are, therefore, of doubtful validity, reliability and usefulness (Zhao and Shen 2008).

Another problem stems from the fact that the number of career guidance professionals currently available is not enough to meet actual demands. Also, among the personnel available, quality is often lacking. According to statistics (Lan and Wang 2010), by 2006, there were more than 26,000 career counselors in China, but the majority of them worked in social organizations, such as Beisen Career, with only a small number in universities. In addition, the distribution of career counselors is not balanced across different regions, with more counselors employed in cities. The employment structure in this field shows a trend toward younger counselors, many of who may lack work experience and may not possess the relevant background knowledge in psychology and pedagogy.

Based on a survey in Beijing, Jin and Fan (2002) found that, on average, there was less than one career teacher for every 1,000 undergraduates in universities. In one context they examined, among the 70 staff engaged in career guidance, only 12.9% possessed a relevant background in career guidance, 45.7% had worked in career guidance for less than 5 years, and only 17.1% had worked for over 10 years.

An additional problem occurs because the concept of ‘career planning’ is not presented explicitly to students early in their university lives. They do not have a clear understanding of what career planning actually entails, and they have not recognized its importance for their own life and future employment (Li 2009).

## Improving Career Guidance

In order to develop effective and adaptive strategies to improve career guidance in Chinese universities, authorities (e.g., Chen 2007; Liu and Li 2007) have pointed out that career guidance and career counseling should not merely try to copy policies and practices from foreign countries. Ideally, Chinese theories and strategies for career guidance and counseling should be created, with due reference to Chinese society's characteristics and practical problems. Indigenous factors that need to be considered include: job market, employment system, educational philosophy, and the social value system (Chen 2007). Since these factors will be distinctive for Chinese communities, local theories and strategies for career guidance and counseling should reflect, or at least acknowledge, these features.

The first step may be to construct a student talent database. This refers to the regular collection of data showing students' study achievements and their continuing progress. When students are first enrolled in colleges, a dynamic database should be set up for each individual (Sun 2009). Wang and Fan (2008) called this approach ‘electro-portfolio assessment.’ They suggest that through analyzing and evaluating the data in the system, teachers can get a comprehensive understanding of students’ development and can guide and support them in accordance with their specific aptitudes and aspirations.

A second improvement suggested by Qian and Fang (2009) is that universities should introduce more participatory and interactive forms of career guidance, such as a ‘Festival of College Students' Career Planning’, including career planning contests, career assessment, interview-style interactive forums, experts and alumni seminars, mock recruitment, and visits to various firms. The influence of peers in affecting students’ behaviors can be utilized positively through a ‘Peer Interdependent Training Course’, which involves university associations, and dormitory, friendship and community groups.

Thirdly, vital to the improvement of career guidance and counseling, is the need to establish a team of career guidance teachers who have high-quality professional knowledge and expertise relevant for college students. The number of professional teachers should be increased, and training of existing staff should be reinforced. Department tutors need to be replaced by full-time guidance teachers for fulfilling the functions of employment guidance. Experts in career counseling from outside college settings should be hired as necessary to supplement existing teaching staff (Ruan 2009).

The existing system of career guidance tends only to emphasize the role of university staff and ignores the impact and importance of family and friends on students’ career choices. Effective career planning systems must be established that include family and parental influences, and also the influence of senior students and classmates. Effective systems should also collect ongoing data and feedback of information regarding the later employment histories of their graduated students (Yao and Zhen 2009). This information can highlight strengths and weaknesses in the career guidance system and can lead to improvements in the content and delivery of information and personal guidance.

Gao (2008) pointed out that career counseling within class is an effective way of providing true contextualized career guidance. It brings career planning into daily teaching activities in class and provides many opportunities for students to ask questions, seek information, and share their concerns and experiences within a social group. University tutors should play an important role in career guidance in collaboration with subject teachers. They can help students acquire comprehensive understanding of curriculum and their major subject in relation to requirements for specific careers (Gao and Huang 2009).

## Conclusion and Implications

In general terms, current research and practice in career guidance and counseling in China remains at an early stage of development, lacking a sound basis in theory, still learning from foreign experiences, and still drawing upon Western methods to a large extent. However, through day-to-day experiences in those institutions where dedicated practitioners are now designing and implementing innovative models, rapid progress is being made and is achieving some positive results.

Based on the literature reviewed in this paper, several suggestions can be made for promoting future research and practical efforts in this domain:

1. Educational systems, policies and practices are deeply embedded in local histories and cultures (Watts *et al*. 2010). In view of unique educational and social situations in China, foreign experiences and models of career guidance and counseling are not fully applicable. Therefore, the key focus of research on career guidance and counseling in the future needs to explore how best to tailor systems and practices specifically to local Chinese contexts.
2. A supportive public policy is critical to the development of career guidance services. Such policy is usually determined largely at national level, though it may be devolved to regional, local or institutional level (Watts 2009; Watts *et al*. 2010). At the national level, career guidance should be supported by policy and resources in order that the development of career guidance can be accelerated. At a local level, based on the different characteristics and needs of students in different regions in China, it is necessary to gain support and assistance from local educational institutions. Researchers should liaise closely with different types of schools, colleges and universities in different regions and share their findings and opinions with career practitioners. This process could help universities design and implement courses that really meet students’ needs.
3. Career counseling, as an indispensable part and strategy of career guidance, needs to be implemented more professionally and made available more widely. As proposed by Watts and Van Esbroeck (2000), there is a general recognition that all counselors will need to use new technologies, including computer-assisted assessment in the diagnostic and self-assessment process, as well as computer-assisted career guidance and counseling via the Internet. In the context of China, the use of new technologies is an extremely positive strategy for integrating and making good use of resources in career counseling.
4. Other Asian regions, such as Singapore, Japan, Hong Kong, and Taiwan, have set up career guidance systems that are sound in terms of local applicability and in their underpinning theory. Through exchanges and dialogue, program designers, researchers and career practitioners in Chinese mainland and in these regions, could gain many useful shared insights in policies, research and practices.

To summarize briefly the main points and implications from this review ― career guidance and career counseling in China started late, but there is now scope for improvement, both in the theory and in practice. Significant progress has been made already in some universities and regions, but overall China still lags behind many other nations. Not only is it necessary to continue to develop strong and effective career guidance services in universities, more action is also required in schools to provide the first steps in preparing youngsters for later career choices and decisions. All forms of career guidance, career counseling, and career assessment in China must take into account indigenous characteristics of the students, and the realities of local and regional employment opportunities. Similarly, indigenous factors need to be considered by researchers investigating or evaluating career services in Chinese mainland settings.
